# Rational use of medicines: How the armed forces do it

**DOI:** 10.4103/0253-7613.71903

**Published:** 2010-12

**Authors:** Sushil Sharma, A.G. Mathur, Sapna Pradhan, D.B. Singh

**Affiliations:** Department of Pharmacology, Army College of Medical Sciences, Delhi Cantt, New Delhi 110 010, India

Sir,

We read with interest the editorial “Rational use of medicines: Achievements and challenges”.[1] published in *IJP* of April 2010. It is indeed disheartening that the practice of Rational use of medicines (RUM) is least visible where it is most needed, especially in developing countries like India. Every effort no matter how small at adopting RUM should be encouraged and lauded. The concept of essential medicines is the cornerstone of any approach which intends to address the issue of RUM.[2] One approach that is being practiced in the Armed Forces Medical Services (AFMS) is the strict adherence to the Priced Vocabulary of Medical Stores (PVMS) list.

PVMS list contains an exhaustive list of drugs and other consumables by their generic names that have been carefully selected on the basis of the latest evidence, ease of availability, and relative cost. Special care is taken to ensure that these drugs are able to treat the entire spectrum of diseases and disorders that may be encountered. The list is divided into 29 sections, each section being devoted to one particular medical/surgical specialty. It is a dynamic list which is updated regularly (approximately every 5 years) by a committee of eminent doctors of AFMS on the basis of evidence based medical literature and the feedback received from defence doctors across the country.

Once the PVMS list is finalized, it is circulated to all the AFMS hospitals across the country with strict instructions to limit their drug procurement and usage to this list. Only under exceptional circumstances, a hospital can deviate from this list. A similar list is also given to the civil hospitals which have been empanelled to treat veterans (Ex-servicemen) of the Armed Forces as a part of the Ex-Servicemen Contributory Health Scheme (ECHS). This encourages rational prescribing practices, prevents the rampant and irresponsible use of medicines under various other influences, while also ensuring uniform standards of care across the hospitals of the AFMS.

## References

[1] Thawani V (2010). Rational use of medicines: Achievements and challenges. Indian J Pharmacol.

[2] WHO Policy Perspectives on medicines-Promoting rational use of medicines: Core components. http://apps.who.int/medicinedocs/pdf/h3011e/ h3011e.pdf.

